# Capability of hypertonic saline cough provocation test to predict the response to inhaled corticosteroids in chronic cough: a prospective, open-label study

**DOI:** 10.1186/1745-9974-9-15

**Published:** 2013-05-20

**Authors:** Heikki O Koskela, Minna K Purokivi

**Affiliations:** 1 Unit for Medicine and Clinical Research, Pulmonary Division, Kuopio University Hospital, PL 1777, Kuopio 70211, Finland

**Keywords:** Chronic cough, Inhaled corticosteroids, Cough provocation tests, Airway hyperresponsiveness, Nitric oxide

## Abstract

**Background:**

Many patients with chronic cough respond to treatment with inhaled corticosteroids but it is difficult to predict which patients are likely to respond. The primary aim of the present study was to define the capability of hypertonic saline cough provocation test to predict the responsiveness to inhaled corticosteroids in chronic cough. The secondary aim was to assess the ability of the saline test to monitor the healing of cough during corticosteroid treatment.

**Methods:**

Forty-three patients with chronic cough were recruited. Before therapy, spirometry, ambulatory peak flow monitoring, nitric oxide measurement, histamine airway challenge, and saline test were performed. Those responding to the first saline test repeated it and the nitric oxide measurement during the subsequent visits. The patients used inhaled budesonide, 400 ug twice daily, for twelve weeks. The treatment response was assessed by Leicester Cough Questionnaire at baseline, and at one, four, and twelve weeks.

**Results:**

Seventy-seven % of the patients demonstrated the minimal important difference in the Leicester Cough Questionnaire indicating a symptomatic response. Neither the response magnitude nor the speed was predicted by the saline test. Histamine challenge showed the strongest predictive ability: The maximal improvement in Leicester Cough Questionnaire total score was 5.08 (3.76 – 6.40) points in the histamine positive and 2.78 (1.55 – 4.01) points in the histamine negative subjects (p = 0.006). Baseline nitric oxide level also associated with the improvement in Leicester Cough Questionnaire total score (p = 0.02). During the treatment, the cough sensitivity to saline gradually decreased among the budesonide responders but not in the non-responders. Nitric oxide levels decreased very rapidly among the responders.

**Conclusions:**

Saline test cannot predict the responsiveness to inhaled corticosteroids in chronic cough but it may be utilized to monitor the effect of this treatment.

**Trial registration:**

The study was registered in ClinicalTrials.gov database (KUH5801112). ClinicalTrials.gov Identifier: NCT00859274

## Background

Chronic cough is reported by 10 – 20% of general population [1]. Current guidelines on chronic cough suggest defining the underlying medical condition and specific treatment directed at it [1-3]. However, often the underlying condition cannot be identified and treatment trials based on the most probable cause are recommended. In about 30% of chronic cough patients there is a corticosteroid-sensitive airway inflammation without objective evidence of asthma. Therefore, a trial of inhaled corticosteroids (ICS) in all patients with chronic cough has been recommended [1]. Given the high number of such patients this leads to a large amount of unsuccessful trials. A test capable to identify patients who are likely to benefit from ICS would be valuable.

Presence of sputum eosinophilia and elevated exhaled nitric oxide (NO) levels have been shown to predict the response to a two-week treatment with ICS in patients with chronic cough [4,5]. A later, retrospective study suggested that elevated NO might even predict a months-long response to ICS in these patients [6]. However, the most recent, prospective study failed to confirm these findings [7]. In that study, neither NO, methacholine challenge, nor adenosine challenge could predict the response to a four-week treatment with inhaled fluticasone 200 ug daily. All the above-mentioned prospective studies may be criticized for short duration and lack of a pre-determined, well validated end point to define a positive clinical response.

Cough provocation tests with hypertonic aerosols are novel types of cough provocation tests. They act via different pathways than the traditional cough tests with capsaicin and citric acid which are capable to stimulate the major sensory receptor for cough, the transient receptor potential vanilloid subfamily member 1 [8]. The cough response to hypertonic aerosols does not involve this receptor [9,10]. The cough sensitivity to hypertonic aerosols is associated with asthma [11-15] and treatment with ICS attenuates the cough sensitivity to them [11]. In asthma, cough sensitivity to hypertonic saline correlates well with the Leicester Cough Questionnaire and with the Juniper’s Asthma Control Questionnaire [16]. Thus, responsiveness to hypertonic saline cough provocation test can be regarded as a valid measure in asthma-associated cough. We therefore hypothesized that this test might predict the responsiveness to ICS treatment in chronic cough. Secondly, we hypothesized that changes in the responsiveness to hypertonic saline during treatment might reflect the healing of cough. The present study was planned to test these hypotheses.

## Methods

### Subjects

Forty-three subjects with chronic cough of at least eight weeks’ duration were recruited using newspaper advertisements. Exclusion criteria were current smoking, any abnormalities in chest x-ray, a febrile respiratory tract infection within six weeks, and a doctor’s diagnosis of asthma. Table 1 shows the characteristics of the subjects.

**Table 1:** The basic characteristics of 43 patients with chronic cough

Age (years)	55.6 (51.9 – 59.4)
Number of females	32 (74%)
Body mass index (kg/m2)	27.4 (25.8 – 29.0)
Number of ex smokers	20 (47%)
Number of atopic patients	14 (33%)
Duration of cough (years)	8.5 (5.5 – 11.6)
Most probable cause of cough^a^	Rhinitis 22 (51%)
	Esophageal reflux 14 (33%)
	Asthma 9 (21%)
Leicester questionnaire total score	13.2 (12.2 – 14.3)
Saline coughs-to-dose ratio (coughs/Osm/kg)	7.24 (5.16 – 9.33)
Number of saline responders	21 (49%)
Histamine response-to-dose ratio^b^ (%/mg)	7.94 (4.74 – 13.3)
Number of histamine responders^c^	21 (50%)
Nitric oxide concentration (ppm)	16.8 (12.7 – 20.9)
FEV_1_ (% of predicted)	93.7 (90.2 – 97.3)
FEV_1_ rise after salbutamol (%)	4.05 (2.86 – 5.24)
PEF variation in ambulatory monitoring (%)	7.13 (5.76 – 8.51)

^a^ Based on the results of the Cough Clinic diagnostic questionnaire. Two patients had more than one most probable cause; ^b^ geometric mean and 95% confidence interval; ^c^ histamine challenge was missed in one patient due to technical reasons.

Five subjects did not complete the study. One discontinued due to suspected budesonide allergy and one due to mild pneumonia. Three subjects did not define a reason for discontinuation. In addition, three subjects were found to have non-satisfactory drug compliance. The 39 subjects who remained in the study for at least four weeks with satisfactory drug compliance were included in the final analysis.

The study was performed in accordance with the Good Clinical Practice guidelines recommended by the Declaration of Helsinki. The study was approved by the institutional Ethics Committee (132//2008) as well as the National Agency for Medicines (EudraCT 2009-009556-21). Written informed consent was obtained from each subject prior to participation in the study.

### Study design

This was a prospective, open-label study. On the first study day the subject was interviewed, the informed consent was obtained, and the Cough Clinic diagnostic questionnaire was filled in [17]. It is a validated questionnaire to assess the most probable cause of cough. Furthermore, a chest x-ray, skin prick tests and histamine airway challenge were performed [18]. The histamine challenge was considered positive if the provocative dose of histamine to produce a 15% fall in forced expiratory volume in one second (FEV_1_) was less than 1.6 mg. The histamine response-to-dose ratio (RDR) was calculated as the final percentage fall in FEV_1_ divided by the final non-cumulative dose of histamine. A peak expiratory flow (PEF) monitoring twice daily was performed during the week between the first and the second study day.

On the second study day the Leicester Cough Questionnaire [19] (LCQ) was filled in, NO was measured according to international guidelines [20] using a chemiluminescence analyzer (Sievers Model 280 NOA; Sievers Instruments, Inc., Boulder, CO, USA), and the hypertonic saline cough provocation test was performed [13]. For the next twelve weeks, the subjects used budesonide inhalation powder 400 ug twice a day (Budesonid Easyhaler, Orion Ltd, Espoo, Finland). The drug compliance was assessed on each subsequent visit utilizing the counter of the inhaler. A satisfactory compliance was defined as more than 50% usage of the prescribed dose.

The third, fourth and fifth study days took place when the subjects had used budesonide for one, four, and twelve weeks, respectively. During these days the LCQ was filled in. In addition, those subjects who were responsive to the first saline test repeated it and the NO measurement during these days.

### Leicester cough questionnaire and the definitions of treatment responses

The Leicester Cough Questionnaire (LCQ) is a 19-item validated, repeatable and responsive questionnaire consisting of physical, psychological and social domains. Answers are graded on 7-point Likert scale which gives a total score ranging from 3 to 21. A small score indicates poor cough-related quality of life [19].

The minimal important difference of LCQ total score is 1.3 points [21]. Documentation of it at any time point during the budesonide treatment was defined as a positive treatment response. The magnitude of the budesonide response was expressed by the maximal improvement in LCQ total score at any time point of the treatment. The speed of the budesonide response was expressed by the time from the start of treatment to the appearance of minimal important difference in LCQ total score, utilizing linear interpolation. If the minimal important difference was never achieved, an arbitrary value of 16 weeks was utilized for statistical purposes.

### Hypertonic saline cough provocation test

The test has been described in detail previously [13]. First, spirometry was performed. Then the subjects inhaled 0.4 mg of salbutamol to prevent bronchoconstriction. Fifteen minutes after the salbutamol inhalation the spirometry was repeated. Then the subject inhaled isotonic phosphate-buffered saline for two minutes via a high-output ultrasonic nebuliser (DeVilbiss Ultraneb 3000, Sunrise Medical Ltd, West Midlands, UK), using tidal breathing. The coughs occurring during the inhalation and two minutes after it were counted up. The number of these “spontaneous” coughs was subtracted from the coughs provoked by each hypertonic solution. Subsequently, they inhaled hypertonic phosphate-buffered saline solutions with osmolalities of 0.6, 0.9, 1.2, 1.5, 1.8 and 2.1 Osm/kg. The challenge was stopped if 15 or more cumulative coughs were recorded (= a positive saline test result). The cough sensitivity to hypertonic saline was expressed as coughs-to-dose-ratio (CDR), calculated as the cumulative number of provoked coughs divided by the final osmolality inhaled.

### Statistical analysis

The sample size requirement for this study was calculated considering clinically relevant a difference of 45% in the proportion of patients responsive to budesonide treatment between the groups with positive and negative saline test result. The calculation was based on previous studies about the prevalence of positive saline tests among patients with chronic cough and the responsiveness of these patients to ICS [7,13]. It was estimated that 42 patients would be required to provide 80% power at the 0.05 level.

The data is expressed as means and 95% confidence intervals. The histamine RDR values and the speed of the budesonide response are expressed as geometric means and 95% confidence intervals. Chi-square test, Friedman’s analysis of variance, Mann–Whitney *U* test and Spearman correlation coefficient were utilized when appropriate.

## Results

### The baseline test results

The patients with asthma as the most probable cause of cough were more sensitive to saline than the rest of the patients (11.2 (6.77 – 15.6) coughs/Osm/kg vs. 6.20 (3.86 – 8.55) coughs/Osm/kg, p = 0.034). There were no other differences in baseline test results between the diagnostic categories (Table 1). Furthermore, there were no significant correlations between LCQ total score or saline CDR and the other tests.

### The response to the budesonide treatment

**Figure 1:**
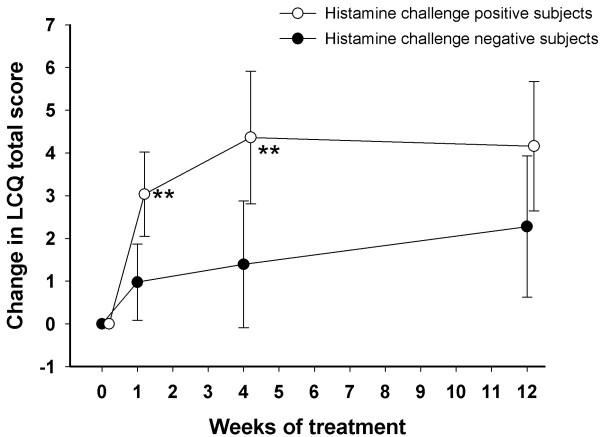
**The changes in Leicester Cough Questionnaire (LCQ) total score during treatment with inhaled budesonide among chronic cough patients who either did or did not respond to histamine challenge at baseline.** ** p < 0.01, Mann–Whitney *U* test.

The mean compliance (the mean amount of inhaled doses in relation to prescribed doses) was 92%. The response was rapid in those eventually responding to the treatment (Figure 1). The minimal important change in the LCQ was achieved by 77% of the subjects. Those with rhinitis as the most probable cause of cough tended to show a larger (p = 0.097) and faster (p = 0.016) response to budesonide than the rest of the patients.

### Baseline tests as predictors of the response to budesonide treatment

The saline test predicted neither the magnitude nor the speed of the budesonide response (Tables 2 and 3). Seventy-five % of the saline test positive and 79% of the test negative subjects demonstrated the minimal important difference in the LCQ total score (p = 0.77). The most important predictor of the treatment response was the histamine challenge result (Tables 2 and 3, Figure 1). NO also showed some predictive value but the spirometric indices and the PEF variability none.

**Table 2:** The responses to the budesonide treatment in 39 chronic cough patients divided by the baseline saline and histamine test results

	**Baseline saline test**			**Baseline histamine challenge**^**a**^		
	**Negative**	**p**	**Positive**	**Negative**	**p**	**Positive**
	**(N = 19)**		**(N = 20)**	**(N = 19)**		**(N = 19)**
Response magnitude (points)	4.45 (2.95 – 5.94)	0.50	3.61 (2.37 – 4.85)	2.78 (1.55 – 4.01)	0.006	5.08 (3.76 – 6.40)
Response speed^b^ (weeks)	1.34 (0.60 – 2.98)	0.72	1.46 (0.68 – 3.15)	2.70 (1.23 – 5.93)	0.008	0.72 (0.36 – 1.43)

Response magnitude was defined as the maximal improvement in LCQ total score at any time point of the study. Response speed was defined as the time in weeks from the start of budesonide treatment to the appearance of minimal important change in LCQ total score.

^a^Histamine challenge was missed in one subject due to technical reasons.

^b^Geometric means and 95% confidence intervals.

**Table 3:** Associations of the baseline test results with the magnitude and the speed of the budesonide response (N = 39)

	**Magnitude of the response**	**Speed of the response**
Saline CDR	Rs = -0.09, p = 0.59	Rs = 0.12, p = 0.45
Nitric oxide level	Rs = 0.37, p = 0.020	Rs = -0.31, p = 0.055
Histamine RDR^a^	Rs = 0.47, p = 0.003	Rs = -0.49, p = 0.002
FEV_1_	Rs = -0.16, p = 0.32	Rs = 0.23, p = 0.17
FEV_1_ rise after salbutamol	Rs = 0.10, p = 0.55	Rs = -0.12, p = 0.48
Mean PEF variation	Rs = 0.18, p = 0.28	Rs = -0.21, p = 0.19

^a^ Histamine challenge was missed in one subject due to technical reasons.

*Rs* Spearman correlation coefficient.

Magnitude of the response was defined as the maximal improvement in LCQ total score at any stage of the treatment. Speed of the response was defined as the time in weeks from the start of budesonide treatment to the appearance of minimal important change in LCQ total score.

### Changes in saline CDR and NO during budesonide treatment and their associations with the changes in LCQ total score

**Figure 2:**
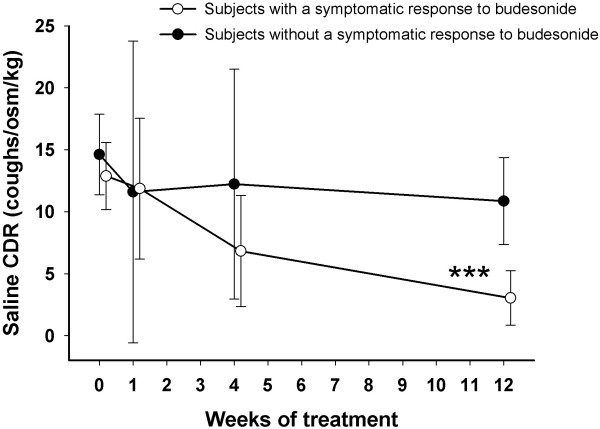
**The cough sensitivity to hypertonic saline during treatment with inhaled budesonide among cough patients who either did or did not show a symptomatic response to the treatment.** The response was defined at each time point as a more than 1.3 points’ increase from baseline in Leicester Cough Questionnaire total score. CDR = coughs-to-dose ratio. *** p < 0.001, Mann–Whitney *U* test.

The saline CDR diminished slowly but significantly, from 13.3 (11.3 – 15.3) coughs/Osm/kg before the treatment to 5.65 (3.14 – 8.15) coughs/osm/kg after the 12 weeks’ treatment (p < 0.001, Friedman’s test). This decline in saline sensitivity was restricted to symptomatic responders (Figure 2). After twelve weeks’ treatment, the saline CDR was significantly lower in the responders than in the non-responders (3.04 (0.84 – 5.24) vs. 10.9 (7.36 – 14.4), p < 0.001). At that stage, the change in saline CDR associated with the change in the LCQ total score (Table 4).

**Table 4:** Associations of the change in LCQ total score with the changes in exhaled NO concentration and saline CDR at various stages of budesonide treatment

**Duration of treatment**	**Change in NO**	**Change in saline CDR**
1 week	Rs = -0.47, p = 0.038	Rs = 0.37, p = 0.11
4 weeks	Rs = -0.40, p = 0.084	Rs = -0.27, p = 0.26
12 weeks	Rs = -0.39, p = 0.11	Rs = -0.45, p = 0.059

*Rs* Spearman rank correlation coefficient.

**Figure 3:**
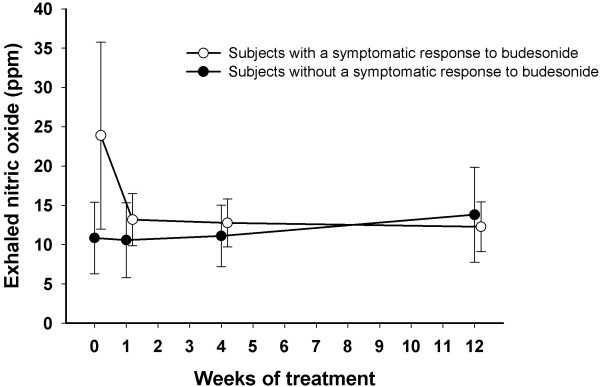
**The exhaled nitric oxide levels during treatment with inhaled budesonide among cough patients who either did or did not show a symptomatic response to the treatment.** The response was defined at each time point as a more than 1.3 points’ increase from baseline in Leicester Cough Questionnaire total score.

There was also a statistically significant change in NO, from 20.0 (11.1 – 28.9) ppm before treatment to 12.8 (10.2 – 15.3) ppm after 12 weeks’ treatment (p = 0.037, Friedman’s test). This decline in NO could be demonstrated only in the symptomatic responders and was very rapid among them (Figure 3). There was a significant association between the drop in NO and a rise in LCQ total score after one week’s treatment (Table 4).

## Discussion

In the present study a positive response to budesonide treatment was defined as the minimal important difference in the highly validated LCQ. This clinically relevant end point was achieved by 77% of the patients corroborating that a substantial proportion of chronic cough patients benefit from ICS [5,7]. However, neither the magnitude nor the speed of the budesonide response could be predicted by the cough sensitivity to hypertonic saline. This was surprising since cough sensitivity to hypertonic aerosols has been shown to associate with asthma in several previous studies [11-15]. Furthermore, the cough patients who were classified as probable asthmatics by the Cough Clinic questionnaire in the present study were more sensitive to saline than the rest of the patients. As asthma is a corticosteroid-sensitive disorder, one would have expected that cough sensitivity to hypertonicity could predict the corticosteroid response. The present negative finding suggests that cough sensitivity to hypertonicity is not directly related to the eosinophilic (corticosteroid-sensitive) airway inflammation. Indeed, there was no correlation between saline CDR and NO in the present study, corroborating our previous findings [13].

Airway responsiveness to histamine was clearly the strongest predictor of the budesonide response. This is contrary to the sole previous study about this issue [7]. In that study, neither methacholine nor adenosine responsiveness could predict the response to a four-week treatment with inhaled fluticasone among 43 patients with chronic cough [7]. This discrepancy is probably due to the fact that only four patients responded to methacholine and two to adenosine in that study. In the present study half of the cough patients responded to histamine. Airway hyperresponsiveness often accompanies chronic cough [18] but its prevalence seems to vary considerably in different cough populations. The patients in the previous study had been referred to special respiratory clinics and those with airway hyperresponsiveness may have been identified already in primary health care level. Instead, the patients of the present study were recruited utilizing newspaper advertisements and enrolled without selection. These findings suggest that measurement of airway responsiveness might be most beneficial at an early stage in the evaluation of chronic cough. Of note, the level of histamine responsiveness in the present cough patients was much milder than that usually found among asthmatic patients [18]. It seems that even mild airway hyperresponsiveness in a cough patient advocates a trial with ICS.

Exhaled NO concentration also showed some value in predicting the response to budesonide. The previous literature about this issue is conflicting [5-7]. This may be due to similar reasons to those discussed in the previous paragraph. If the cough population contains very few subjects with elevated NO, it cannot predict the response to ICS [7].

The subjects who showed the greatest benefit from inhaled budesonide in the present study were often classified as rhinitic patients by the Cough Clinic diagnostic questionnaire [17]. One explanation for this unexpected finding may be that the ability of this questionnaire to identify the underlying reason for chronic cough is worse than anticipated. Perhaps a more likely explanation is that chronic rhinitis and asthma can be regarded as manifestations of the same disorder (the one-airway hypothesis) [22]. Among patients with chronic cough and symptoms suggestive of either rhinitis or asthma, an interesting management option might be a concomitant treatment with both nasal and inhaled corticosteroids, or administration of inhaled corticosteroid through the nose [23].

The second aim of the present study was to evaluate saline test and NO measurement in their capability for monitoring the effect of ICS therapy in chronic cough. These two biomarkers behaved very differently in this respect.

After just one week’s therapy a drop in NO levels could be demonstrated among patients with a positive treatment response. This corroborates previous findings among asthmatics that NO levels very rapidly respond to ICS and highlights the high sensitivity of eosinophilic inflammation to corticosteroids [24]. The decrease in NO reflected the healing of cough because it could only be seen in the budesonide responders and the changes in NO showed an association with the changes in LCQ total score after one week’s treatment. These findings, together with previous studies [4,5], suggest that NO best predicts the short-term response to ICS in chronic cough.

Saline CDR, in turn, decreased slowly. The difference between responders and non-responders was statistically significant not until 12 weeks’ treatment. The decrease in saline CDR also reflected the healing of cough because it could only be seen in the budesonide responders and the changes in CDR showed an association with the changes in LCQ total score after 12 weeks’ treatment.

The time course of the decrease in saline cough responsiveness was similar to the attenuation of the cough responsiveness to mannitol during treatment with ICS [11]. Mannitol is another hypertonic agent. In asthma, the decrease in the cough responsiveness to mannitol is apparent after three months’ ICS therapy but continues to decrease up to six months. The decrease in cough responsiveness to mannitol associates significantly with the healing of cough [11]. ICS may also affect the cough sensitivity to capsaicin and citric acid but the results are conflicting [25-28]. The decrease in cough responsiveness to hypertonic aerosols during ICS treatment possibly represents a slow de-sensitization of the airway sensory nerves as the levels of inflammation- and oxidative stress-associated mediators, capable to sensitize sensory nerves, decrease [29,30].

The main limitation of the present study is its uncontrolled nature. The large proportion of budesonide responders may be partly explained by the placebo effect and/or the regression-to-mean phenomenon. The latter may be unlikely because the subjects had suffered from cough for a very long time, mean 8.5 years. The main strengths of the present study are, compared to the previous prospective studies about this issue [4,5,7], the well validated indicator of the treatment response and the longer duration of the treatment. The non-selected population may also be regarded as strength.

## Conclusions

The present study confirms that a large proportion of patients with chronic cough benefits from ICS treatment. However, the responsiveness to ICS cannot be predicted by the hypertonic saline cough provocation test. In the present non-selected cough population, responsiveness to histamine airway challenge was the strongest predictive feature of the ICS response. Both the saline test and NO measurement might be used to monitor the effect of an anti-inflammatory treatment in chronic cough. These tests probably measure different pathophysiological phenomena and therefore, provide complementary information about the healing of cough.

## Abbreviations

CDR: Coughs-to-dose ratio; FEV1: Forced expiratory volume in one second; ICS: Inhaled corticosteroids; LCQ: Leicester cough questionnaire; NO: Exhaled air nitric oxide concentration; PEF: Peak expiratory flow; RDR: Response-to-dose ratio.

## Competing interests

Heikki Koskela owns Orion Ltd, Finland, shares worth 9000 euros.

Minna Purokivi has no competing interests.

## Authors’ contributions

HK mainly designed the study, analyzed and interpreted the data, and wrote the manuscript. MP helped to design the study, partly analyzed the data, and revised the manuscript critically for important intellectual content. Both authors have read and approved the final version of the manuscript.
